# Risk factors for early colonization of mutans streptococci – a multiple logistic regression analysis in Swedish 1-year-olds

**DOI:** 10.1186/1472-6831-14-147

**Published:** 2014-12-03

**Authors:** Ann Ingemansson Hultquist, Peter Lingström, Mats Bågesund

**Affiliations:** Public Dental Service/Folktandvården, Stora Trädgårdsgatan 39, SE 593 42 Västervik, Sweden; Department of Cariology, Institute of Odontology, Sahlgrenska Academy, University of Gothenburg, Box 450SE-405 30, Göteborg, Sweden; Center for Orthodontics and Pediatric Dentistry, Public Dental Service Östergötland, Folktandvården Druvan, SE 601 82 Norrköping, Sweden; Division of Pediatrics, Department of Clinical and Experimental Medicine, Faculty of Health Sciences, Linköping University, SE 581 85 Linköping, Sweden

**Keywords:** Children, Dental caries, Food and beverages, Infants, Risk factors, Sibling, Streptococcus mutans, Toothbrushing, Tooth eruption

## Abstract

**Background:**

Mutans streptococci (MS) are closely related to the development of dental caries and are usually established in the oral cavity during early childhood. The aim of the study was to identify factors associated with the presence of MS in Swedish 1-year-olds.

**Methods:**

Parents completed a questionnaire on different caries-associated factors and an oral bacterial sample was collected from 1,050 (526 boys, 524 girls) 1-year-olds. Multiple logistic regression analyses were performed to identify risk factors for colonization with MS.

**Results:**

MS were found in 27% of the 1-year-olds with teeth. High or very high MS scores (2–3) were found in 72 (7%) of the children. MS score was correlated to the number of erupted teeth (p < 0.001). No difference due to gender was found. Multiple logistic regression analysis showed that presence of bacteria was associated with: *caries in a sibling*, *other beverages than water between meals*, and *more than 8 erupted teeth*. High or very high MS scores (2–3) were associated with *other beverages than water between meals*, and *more than 8 erupted teeth*.

**Conclusions:**

Number of teeth present, diet and family aspects were factors associated with presence of MS in 1-year-olds. To develop high or very high MS scores, the number of erupted teeth and dietary habits are important.

## Background

A reduced caries incidence among children has been observed in the Nordic countries during the last decades [1, 2], but dental caries is still a severe problem for those who are affected.

Due to the short distance from the outer dental surface to the pulp in the primary teeth, the progression of dental caries can be fast in early childhood [3] and the risk for dental infection is obvious. The risk for spreading of dental infections due to caries disease, which in severe cases can cause life threatening conditions [4], makes it important to prevent dental caries at an early age. The problems associated with dental treatment in early childhood - related to the infant’s inability to understand the necessity for dental treatment, the limited ability to cooperate to the often unpleasant dental treatment and the following risk for development of dental fear - makes it even more urgent to avoid the establishment of dental caries among the youngest children [5].

Several caries associated risk factors have previously been identified [6, 7], among which the oral colonization of mutans streptococci (MS) is a factor closely related to the development of dental caries both among children and adults [7–11].

MS from dental plaque has been associated to dental caries in preschool children [12, 13] and higher MS scores have been found correlated to the caries incidence both in 6- to 7-year-olds [14] and in preschool children [15]. Children with a higher MS score can therefore be considered to have a higher caries risk than those without colonization of MS or those with a lower MS score.

Although MS is not naturally present in the oral cavity from birth [16] colonization often begins around the time point when the first teeth erupt [17, 18]. It has been shown that the timing of MS colonization in the oral cavity influences caries risk [19, 20], it is therefore considered important to identify factors associated with oral MS colonization at an early age. The eruption of teeth has been considered as a contributing factor for the colonization of MS in the oral cavity [17, 18] and as the timing of eruption of teeth is similar among boys and girls [21, 22] at the same time as studies have found a higher caries prevalence among boys [23], we found it interesting to evaluate how eruption of teeth and gender may influence the colonization of MS among infants.

Neglected tooth brushing is known to contribute to the growth of bacteria [24] and since oral hygiene routines are considered to be better among girls than among boys (at least among primary school children) [25], we found it relevant to evaluate the influence of tooth brushing and gender on the colonization of MS in the oral cavity.

The aim was to evaluate possible risk factors associated to early oral colonization of MS in 1-year-olds, and the more specific hypotheses were that: 1) MS is not present in the oral cavity before the first tooth erupts, and 2) boys have higher MS scores than girls at one-year of age.

## Methods

### Patients

All 1-year-old children who lived in the region served by the Public Dental Clinic Vidablick, Norrköping, Sweden, between 2001 and 2010 were invited to participate in the study. Dated invitations were sent by mail to the parents around the time point for the 1-year birthday of their child. Approximately 130 invitations were sent each year. Those who missed the appointment without cancelling received no new invitation while those who cancelled the visit in advance were rescheduled for a new appointment.

All children who had taken antibiotics within the previous 2 weeks were given a new appointment a few weeks later. Approval of the study was obtained from the Regional Ethical Review Board at Linköping University.

### Procedure

A questionnaire on caries-associated and other pertinent factors was sent to parents together with an invitation to the clinic. Parents were informed not to brush the child’s teeth the morning before the visit. Informed consent was obtained from participating parents or legal guardians.

The questionnaire (Table 1) was completed by the parents. After answering the questionnaire and clinical sampling, parents received information on oral health including how to avoid dental caries and how to handle dental trauma.

**Table 1:** **Items from the questionnaire and abbreviations**

Item	Abbreviation
Does the child have any siblings?	*Sibling*
Have any of the siblings had dental caries?	*Caries in sibling*
Does the child eat or drink anything except water at night?	*Night meal*
Is the child still breastfed?	*Breastfed*
Does the child have any illness/disease?	*Disease*
Does the child regularly take any medication?	*Medication*
Does the child drink anything except water between the meals?	*Beverage other than water*
How many teeth does the child have?	*Erupted teeth*
Do you brush the child’s teeth?	*No tooth brushing*

A visual inspection of the front teeth was done to evaluate visible signs of dental plaque. One bacterial sample per child was collected using a Quick-Stick® (Dentsolv AB, Saltsjö-Boo, Sweden). If any tooth had erupted, the sample was taken from the buccal tooth surface/s of the (preferably maxillary) incisors close to the marginal gingival sulcus. If no teeth had erupted, the sample was taken from the tongue and cheek. Plaque on the Quick-Stick was transferred to an incubation strip (Dentocult® Strip Mutans test; Orion Diagnostica, Espoo, Finland) and cultivated according to the manufacturer’s instructions The sample was incubated in a heat chamber (Memmert GmbH, Hannover, Germany) at the clinic for 48 hours at 37°C and then analyzed. The numbers of adherent colonies were compared with a chart supplied by the manufacturer and given a score between 0 and 3 to indicate low to high levels of MS (Table 2). An MS score of 0 included all MS bacterial samples with less than 10^4^ CFU/ml.

**Table 2:** **Characteristics of the 1-year-olds and possible difference between the genders**

Value	Boys	Girls	Total/all	Difference
	N = 526	N = 524	N = 1,050	p-value
Age at visit (months; mean ± S.D.)	13.4 ± 1.5	13.6 ± 2.0	13.5 ± 1.8	0.11^¤^
Age range: min-max (months)	11-20	11-21	11-21	
Number of teeth (mean ± S.D.)	8.3 ± 4.0	7.8 ± 4.2	8.0 ± 4.1	0.04^¤^
Number of teeth (range)	0-20	0-20	0-20	
MS growth	150 (29%)	138 (26%)	288 (27%)	0.43^¤¤^
MS score:				
0 (<10^4^ CFU^#^/ml)	394 (75%)	394 (75%)	788 (75%)	0.8095^¤¤^
1 (>10^4^ < 10^5^ CFU^#^/ml)	95 (18%)	95 (18%)	190 (18%)	
2 (>10^5^ < 10^6^ CFU^#^/ml)	23 (4%)	23 (4%)	46 (4%)	
3 (>10^6^ CFU^#^/ml)	14 (3%)	12 (2%)	26 (2%)	

^#^CFU = colony-forming unit.

Statistical significance of difference was evaluated using:

^¤^Student’s *t*-test for continuous variables.

^¤¤^Chi-square test for comparison of proportions.

Only one dental hygienist or dental assistant was present at each visit. Altogether four persons (one dental assistant with more than 30 years’ experience and three dental hygienists with more than 3 years’ experience) handled the information and collected the bacterial samples throughout the study.

### Statistical analyses

Student’s t-test and chi-square analysis was used for comparison of groups depending on whether the variables were continuous or categorical. The Spearman rank correlation analysis was used to analyze categorical data and continuous variables. In the multiple logistic regression analysis the variables were dichotomized before inclusion in the analysis. Multiple logistic regression analysis was performed to identify which variables were associated to the presence of MS or to high or very high (2–3) MS score. A p-value <0.05 was considered statistically significant. All statistical analyses were done with Stata/MP (version 12.1, StataCorp LP, College Station, TX 77845, USA).

## Results

### Study group

Parents of 1,121 children (566 boys, 555 girls) accepted the invitation and visited the clinic. The parents of 44 children (23 boys, 21 girls) did not complete the questionnaire. In another 27 (13 boys, 14 girls) children, the bacterial test could not be completed. Thus, only subjects with complete information were included in the further analyses. Children without siblings were included in the analysis.

Table 2 presents characteristics of the 1,050 (526 boys, 524 girls) children who completed the questionnaire and bacterial test. Despite the finding that the boys had a higher number of erupted teeth than the girls, the age of the children did not differ significantly between the genders (Table 2).

### Presence of mutans streptococci

MS growth was found in 288 (27%) of the 1,050 children. Table 3 lists the factors associated with a presence of bacteria according to the chi-square test. MS was significantly (p < 0.05) more often present in the oral cavity among the 1,033 children with erupted teeth (28%) than among the 17 children without erupted teeth (6%). Only 1 of the 17 children with no erupted teeth presented any growth of MS. Presence of MS was significantly (p < 0.01) more frequently found among the 342 children with more than 8 teeth erupted (33%) than among the 708 children who had fewer than 8 teeth erupted (25%). No significant difference for MS in relation to gender was found (Table 2). Other factors associated with the presence of MS were *caries in sibling* (p < 0.01), intake of other beverage than water between the meals (p < 0.001) (Table 3). According to the multiple logistic regression analysis, the variables *caries in sibling* (p < 0.05), *other beverage than water* between the meals (p < 0.001), and *more than 8 erupted teeth* (p < 0.05) were factors associated with the presence of MS (Table 4).

**Table 3:** **Chi-square analysis of factors possibly associated to oral mutans streptococci in 1-year-olds**

Value	Positive/number of observations	Chi-square test MS growth		Chi-square test MS score 2–3	
	(N = 1,050)	(N)	p-value	(N)	p-value
*Caries in sibling* ^*^	114/576	(42)	0.003	(12)	0.01
*Caries in sibling* ^**^	114/1050	(44)	0.02	(32)	0.10
*Night meal*	338/1040	(95)	0.65	(33)	0.01
*Breastfed*	121/1048	(37)	0.40	(12)	0.16
*Disease*	32/1050	(9)	0.93	(2)	0.89
*Medication*	21/1045	(8)	0.26	(1)	0.70
*Beverage other than water*	295/1038	(108)	<0.001	(32)	0.002
*>0 erupted teeth*	1033/1050	(287)	0.04	(72)	0.26
*>8 erupted teeth*	342/1050	(112)	0.007	(34)	0.006
*No tooth brushing*	63/1050	(23)	0.096	(7)	0.17

^*^Only children who had siblings.

^**^All children, whether or not they had siblings.

Factors associated with the presence of mutans streptococci (MS) and with high or very high (2–3) MS score in 1-year-olds according to Chi-square test. For explanation of the abbreviations of the values, see Table 1.

**Table 4:** **Multiple logistic regression analysis to analyze factors possibly associated to the presence of mutans streptococci in 1-year-olds (N = 1,038)**

Value	Odds ratio	95% CI	p-value
*Caries in sibling*	1.60	1.05–2.45	0.028
*Night meal*	0.97	0.70-1.34	0.85
*Breastfed*	1.21	0.76-1.92	0.42
*Disease*	0.78	0.30-2.00	0.60
*Medication*	1.64	0.54-4.99	0.38
*Beverage other than water*	1.75	1.30–2.37	<0.001
*>0 erupted teeth*	7.68	0.95–62.34	0.056
*>8 erupted teeth*	1.44	1.07–1.94	0.015
*No tooth brushing*	1.62	0.88–2.99	0.12

For an explanation of the abbreviations of the values, see Table 1.

### MS score

A high or very high MS score occurred in 72 (7%) of all examined children (Table 2). Factors associated with a high or very high MS score according to the chi-square test were *caries in sibling* (p < 0.01), *night meal* (p < 0.01), *other beverage than water* between the meals (p < 0.01) and *more than 8 erupted teeth* (p < 0.01) (Table 3). According to the multiple logistic regression analysis (Table 5) association with a high or very high MS score (2–3) was found for *other beverage than water* between the meals (p < 0.05), and *more than 8 erupted teeth* (p < 0.01).

**Table 5:** **Multiple logistic regression analysis of factors possibly associated with high or very high (2–3) mutans streptococci score in 1-year-olds (N = 1,028)** ^**¥**^

Value	Odds ratio	95% CI	p-value
*Caries in sibling*	1.37	0.70–2.70	0.35
*Night meal*	1.66	0.98-2.82	0.061
*Breastfed*	1.23	0.60-2.54	0.57
*Disease*	0.78	0.15-4.01	0.76
*Medication*	0.71	0.07-6.79	0.77
*Beverage other than water*	1.91	1.16–3.14	0.011
*>8 erupted teeth*	2.06	1.25–3.40	0.005
*No tooth brushing*	1.57	0.67–3.73	0.30

^¥^Including children who had no siblings.

None of the pre-dentate children had a high or very high MS score, why the variable (*>0 erupted teeth*) was excluded from the analysis. For an explanation of the abbreviations of the values, see Table 1.

The MS score was relatively low (MS score 1) in the child who presented MS growth of bacteria before eruption of the first tooth. Table 5 lists results from the multiple logistic regression analysis of variables associated with a high or very high MS score (2–3). The Spearman rank correlation test found a correlation between number of teeth and MS score (p < 0.001, Spearman’s rho = 0.1062).

Most of the variables that were significantly associated with the presence of MS or with a high or very high MS score in the chi-square test (Table 3) were also significant in the multiple logistic regression analyses (Tables 4 and 5).

## Discussion

### Study group

The socioeconomic status of the population living in the region served by the Public Dental Clinic where the study was performed is considered to be representative for the region. From the population distribution in this area, about 85% of the invited children aged around 1 year participated. According to instructions from the Swedish Ethical Boards “it must be clarified that participation in research projects is voluntary and that one is entitled to withdraw at any moment without giving any explanation” [26]. We did therefore not investigate reasons for drop out. One reason for non-attendance at the 1-year appointment may be that parents, who had previously attended a 1-year-appointment with an older child where caries risk had been assessed to be low, may have considered it less important to attend a second appointment with their younger child, since the focus of the visit was information.

A lower attendance rate at the dental clinic has been found to be higher in families with for example low socioeconomic status [27], language problems, or parental dental fear [28, 29]. This study did not include socioeconomic status, immigrant background, parents’ dental fear or parents’ level of MS in the evaluation. This should be considered when interpreting the results from the study.

### Data collection procedure

The four experienced professionals who collected the bacterial samples and other data all had the same information and collected the data in a standardized manner. The four professionals were calibrated regarding examination procedures and bacterial sample evaluation. Bacterial sample cultivation was performed according to the manufacturer’s instructions. Since the start of the study, other published studies have argued for relevance of including samples from the tongue for the detection of MS [30]. We anyhow decided to continue our data collection as planned since the start of the study with sampling from teeth, and only if no teeth were erupted the sample was taken from the tongue and cheek, as described in the Methods section.

### Presence of mutans streptococci

The finding that 73% of the 1-year-old children not exhibited any MS growth while 75% of the children with positive findings had an MS score of 0 (Table 2) is explained by the definition of the MS score 0, which includes all MS bacterial samples with less than 10^4^ CFU/ml (Table 2), according to instructions from the manufacturer.

Even if the major intra-familial transmission of bacteria is vertical [31], the finding that the variable *caries in sibling* was associated with presence of MS is supported by previous findings of horizontal transmission of MS between unrelated preschool children [32–34]. Thus, the association between growth of MS at 1-years-age and *caries in sibling* found in our study could be the result of horizontal transmission [32, 34] from a sibling or by vertical transmission from the mother or father to the child [31], or a combination of these two. A previous study [34] supports the possibility for different ways for transmission of MS.

The association of high or very high (2–3) MS score with the variables *night meal* (according to the Chi-square test) and consumption of *beverages other than water* between meals (according to both Chi-square test and multiple regression analysis) are in agreement with Thorild et al. [35], who found that daily intake of candy and sugar drinks between meals in feeding bottles were associated with MS prevalence in children aged 1.5 and 3 years. These findings are also in agreement with the findings of Grindefjord et al. [8], who performed a study on MS among 1000 1-year-old children in the Stockholm area, and found an association between the presence of MS and sugar-containing beverages at night and total consumption of sugar-containing beverages at 1-years-age. Our finding that *no tooth brushing* was not significantly associated with the presence of MS is in contrast to the results presented by Zhou et al. [36], who found that colonization of MS in 8 to 32-month-old children was associated with poor oral hygiene [36].

### Tooth eruption and presence of MS

The association found between tooth eruption and presence of MS in the Chi-square test (Table 3) agrees with previous findings where MS was cultivated from bacterial colonies sampled from the tongues of 1-year-olds [8]. This association was anyhow not significant in the multiple regression analysis, even if the odds ratio for erupted teeth was higher than for the other variables (Table 4). It has been postulated that MS is unable to colonize the oral cavity until part of the first primary tooth has erupted [37]. In our study, only one child presented growth of MS before eruption of the first tooth. Since the number of teeth were evaluated by the parents (in a questionnaire) and not in the clinical examination, it is possible that a minor part of a tooth had been exposed to the oral cavity before it was observed by the parents. Several studies, however, have shown that colonization of MS is possible also prior to eruption of a tooth surface [18, 29, 38]. The association between number of teeth and MS score and the finding that more than 8 erupted teeth made MS colonization more frequent is probably due to the larger tooth surface area available for MS colonization in a more developed dentition. This is particularly evident when the primary molars have erupted [8, 39]. It’s also possible that the time since the first tooth erupted (age of the child) rather than the presence or absence of teeth could have contributed to some of the differences found regarding MS colonization between the groups of children with different number of teeth erupted. The first hypothesis – that MS is not present in the oral cavity before the first tooth erupts – was rejected, since it was possible to identify MS before eruption of the first tooth. It can be discussed whether the 17 pre-dentate children should have been excluded from the study, since they had no erupted teeth and the sampling in these patients were performed from the oral mucosa instead of from the tooth surface. Excluding the pre-dentate children would have made evaluation of the first hypothesis impossible. A complementary analysis of the results excluding the 17 pre-dentate children, anyhow, resulted in almost identical results, why we decided to include them in the analysis.

The finding that *no tooth brushing* was not significantly associated to presence of MS in the multiple logistic regression analysis is in contrast to other studies on preschool children both before [22] and after 1-years-age [40].

### MS score

The finding that 7% of the samples from 1-year-old children in the present study had a high or very high MS score (2–3) is in agreement with the findings of Grindefjord et al. [8] who found a high or very high MS score in bacterial samples from the tongue among 6% of 1-year-olds in the Stockholm area.

The finding that the boys had a higher number of erupted teeth than the girls is not supported by previous studies among Swedish [21] and Finnish children [22] where the mean age at the eruption of the first primary tooth was similar for the boys and the girls. But even if the number of erupted teeth in our study was higher among the boys, no significant difference regarding MS score was found between the boys and the girls. This study did not focus on the time since eruption, which may be of higher importance. The second hypothesis – that boys have higher MS scores than girls at one-year age – was rejected.

The findings from the present study are important for all health professionals, who are interested in the multi-factorial etiology of MS colonization of the oral cavity. It will provide a better knowledge and possible implications for the clinician, who wants to provide a better preventive dental care to avoid dental caries in pre-school children.

## Conclusions

Factors associated both with the presence of MS and with a high or very high MS score at 1-years-age are *caries in a sibling*, consumption of *beverages other than water between meals*, and *more than 8 erupted teeth*. The number of erupted teeth, dietary habits and family factors are important for the colonisation of MS in the oral cavity.

## Abbreviations

MS: Mutans streptococci
CFU: Colony-forming unit.
